# Supplementary material for: A Fast and Efficient Ensemble Transfer Entropy and Applications in Neural Signals
Source: Entropy (Basel). 2022 Aug 13;24(8):1118. doi: 10.3390/e24081118 (PMC9407540; doi:10.3390/e24081118)
Supplement: Supplementary file 1 [file entropy-24-01118-s001.zip › entropy-1764252-supplementary.pdf]

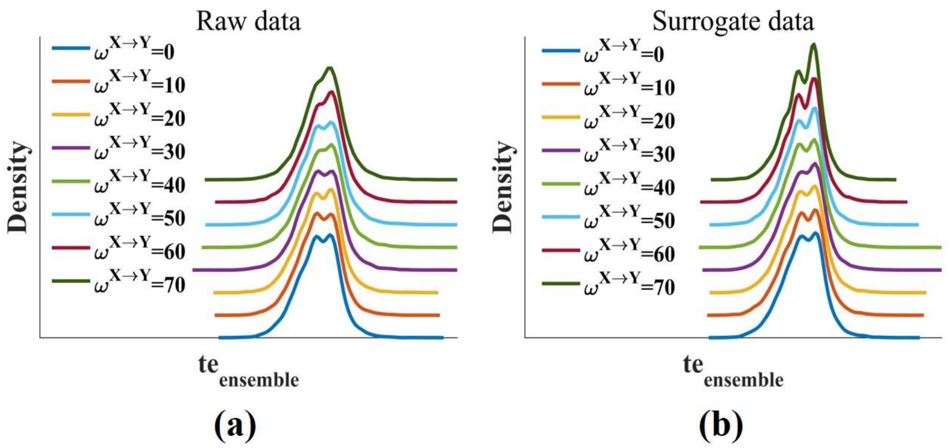

Figure S1. The distributions of $te_{ensemble}$ values which were estimated from the raw and the surrogate data with varied $\omega^{X\to Y}$. The $te_{ensemble}$ values of the raw and the surrogate data were non-normally distributed.

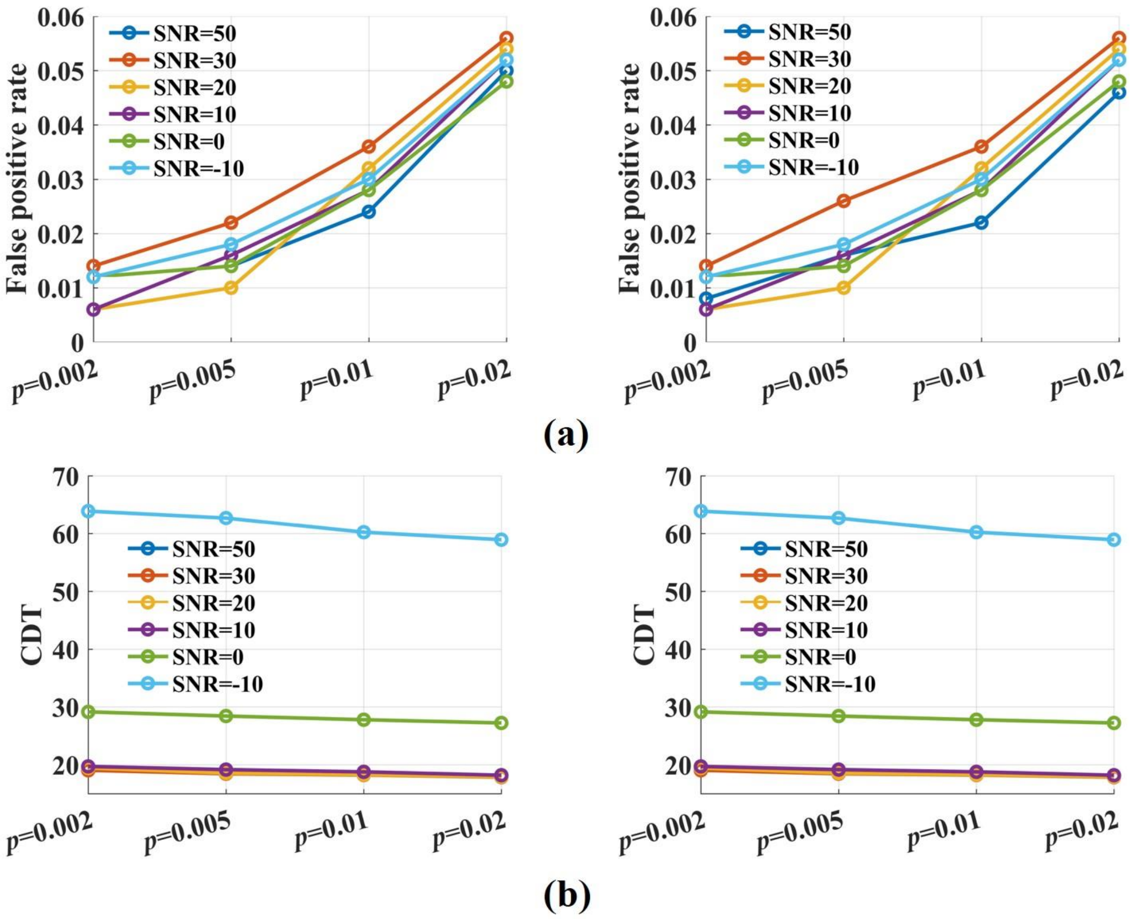

Figure S2. The false positive rate and the CDT values of the novel $TE_{ensemble}$ with the t-test and the Wilcoxon rank sum test (left: t-test, right: Wilcoxon rank sum test). **(a)** The false positive rates of the novel $TE_{ensemble}$ with two statistical methods were almost the same. They all clustered at 0.01 when p was 0.002 and increased to 0.05 when p was 0.02. **(b)** The CDT values of the novel $TE_{ensemble}$ with the Wilcoxon rank sum test were coincided with those of the novel $TE_{ensemble}$ with the t-test.
